# A Review of the Strain Diversity and Pathogenesis of Chicken Astrovirus

**DOI:** 10.3390/v9020029

**Published:** 2017-02-10

**Authors:** Victoria J. Smyth

**Affiliations:** Agri-Food and Biosciences Institute, Stormont Laboratory, Stoney Road, Belfast, BT4 3SD, UK; victoria.smyth@afbini.gov.uk

**Keywords:** chicken astrovirus, strain diversity, pathogenesis

## Abstract

Although a relatively recently emerged virus, identified only in 2004 as a separate species of avian astrovirus, chicken astrovirus (CAstV) has been associated with poor growth of broiler flocks, enteritis and diarrhea and is a candidate pathogen in cases of runting stunting syndrome. More recently CAstV has been implicated in cases of two other diseases of broilers as the sole etiological agent, namely severe kidney disease of young broilers with visceral gout and the “White Chicks” hatchery disease. Examination of the strains of CAstV associated with the two latter diseases reveals they are closely related genetically. This review will discuss the pathogenesis of CAstV in relation to strain diversity and the effects of vertical versus horizontal transmission, virus load, co-infections and age of bird at infection, all factors that may impact upon disease severity.

## 1. Introduction

Chicken astrovirus (CAstV) is a recently emerged virus and the most recently identified member of the avian astroviruses. With a shared familial morphology and genomic arrangement, like other astroviruses CAstV is a small, round, nonenveloped virus typically <35 nm in diameter with a positive sensed, single-stranded RNA genome that, being close to 7.5 kb in length [1], is within the astrovirus family genome size range of 6.2 kb (human) to 7.7 kb (duck) [2]. Astroviruses primarily cause enteric infections and infect many animal species including humans, where they are a leading cause of infant diarrhea. Madeley and Cosgrove termed these viruses astroviruses, from the Greek word astron, meaning “stars”, due to the protruding capsid spikes that give the characteristic star-like appearance under electron microscopy [3]. Astrovirus species infecting mammals are classified into the genus *Mamastrovirus*. The other major group of astroviruses that have been studied are the avian astroviruses, particularly those that infect commercial flocks although they are also detected in wild birds, and are classified in the genus *Avastrovirus*, which along with *Mamastrovirus* make up the two genera of the family *Astroviridae*.

Historically astroviruses have been named according to the species they infect, e.g., turkey astrovirus (TAstV) although species cross-over has been observed for some astroviruses, e.g., astroviruses of chickens have been detected in turkeys [4]. Officially, there are three different astrovirus species that currently comprise the *Avastrovirus* genus according to the International Committee on Taxonomy of Viruses, namely *Avastrovirus 1, 2* and *3* (Table 1), in keeping with the naming of mammalian astroviruses as *Mamastrovirus 1–19*. Although the first report of avian disease caused by astroviruses was in ducklings in 1965 [5], the virus responsible was only recognized as an astrovirus in the mid-1980s by electron microscopy [6] and is referred to as duck hepatitis virus 2 (DHV-2) in older papers and as duck astrovirus serotype I (DAstV-1) more recently [7]. A second astrovirus of ducks, originally called DHV-3 also causes hepatitis in ducklings [8]. It is now known as DAstV-2 and is antigenically and genetically distinct from DAstV-1 [9]. Two astrovirus species were isolated from turkeys: the first was identified in the UK in 1980 and called TAstV serotype 1 (TAstV-1) [10] and the second species, TAstV-2, was reported in 2000 [11].

There are two astrovirus species that infect chickens and both are associated with growth problems, enteritis and kidney lesions in young chickens. The first, avian nephritis virus (ANV), was isolated from a one week old, normal broiler chick in 1976 [12] and was originally thought to be a picornavirus [13], but later identified as an astrovirus [14]. The second astrovirus of chickens is called chicken astrovirus (CAstV) and is a separate species from ANV. Recent research has revealed that CAstV infections are highly common in broiler chickens and have strong associations with diseases of young birds and hatchery disease, that is disease which occurs prior to or during hatch, which will be discussed within this review in the context of genetic variation.

## 2. Identification and Genomic Structure of Chicken Astrovirus

In 2004 three isolates were cultured from two submissions of broiler chicks with runting stunting syndrome (RSS) and one submission from a flock with uneven growth. The isolates were antigenically identical [15]. Genetic sequencing of a 320 base pair reverse transcription (RT)-PCR amplicon made from RNA extracted from one of the isolates showed the agent was related to TAstV and ANV but was from a separate species also identified as an astrovirus and termed “chicken astrovirus” [15]. Prior to its molecular identification in 2004, CAstV had been described as an “enterovirus-like virus” (ELV) due to sharing similar characteristics to viruses of the genus *Enterovirus* of the family *Picornaviridae* [16,17].

Sequencing of the CAstV genome has shown that it shares a similar genetic organisation to other astroviruses being composed of only three open reading frames (ORF). The first two ORFs, ORF 1a and ORF 1b, code for non-structural proteins including a protease (ORF 1a) and an RNA dependent RNA polymerase (ORF 1b). In keeping with other astroviruses, CAstV contains a conserved heptameric frameshift motif at the 3′ end of ORF 1a [18] that is involved in the translation of ORF 1b in a different frame to ORF1a although possibly through a different mechanism to other astroviruses [1]. The third ORF, ORF 2, codes for the capsid protein, the most variable region of the genome, especially in the 3′ half of the ORF which codes for the outer surface of the capsid including the star-like capsid spikes which interact with the host immune system hence variability is desirable. It also contains the start of the conserved s2m motif [19], which continues into the 3’ untranslated region (UTR). A short 5′ UTR exists upstream of ORF 1a and a longer 3′ UTR after ORF 2. A polyadenylated tail is located at the extreme 3′ region completing the positive sensed, single stranded RNA genome.

## 3. Infection, Transmission and Strain Diversity of Chicken Astrovirus

CAstV is an enteric pathogen and infections often occur very early, either transmitted horizontally by the fecal–oral route, or some CAstV strains can also be vertically transmitted from naive in-lay parent birds, and chicks may hatch shedding high levels of CastV. CAstV is more resistant to disinfection and cleaning than other viruses as it is non-enveloped and may be more persistent in poultry houses where darkling beetles can act as vectors for CAstV [20]. For instance, CAstV was detected in internal tissues and in washings from the surface of darkling beetles by RT-PCR [21]. A recent investigation was carried out into CAstV carryover contamination between broiler crops in commercial broiler houses after the removal of spent litter both before and after cleaning and disinfection using proprietory disinfectants at recommended concentrations [21]. Preliminary results showed that ~1–2 log reductions in CAstV levels were typically achieved when detected by quantitative RT-PCR at ten locations in UK broiler houses including feeders, floors, sills and walls, where CAstV levels became extremely low but even when fumigation was part of the cleaning regime, newly placed chicks quickly became horizontally infected shedding moderate levels of CAstV by day 4. Although the majority of these chicks were not shedding CAstV at day 0, it is possible that CAstV horizontal infection could have occurred prior to placement in the broiler houses.

CAstV infections usually occur within the first days or week of life, and, and the earlier they are contracted, especially vertical infections, may result in a worse outcome, although this will depend on the particular CAstV strain, since, as is typical of viruses with RNA genomes, they vary widely in pathogenicity. Also the viral load (dose) at the time of infection and the presence of maternal antibodies against CAstV will impact on the development of disease. Other important factors include the presence of other enteric pathogens such as ANV, which is frequently detected in co-infections with CAstV, and also avian orthoreoviruses and fowl adenoviruses, to name some of the more ubiquitous enteric viruses often found in co-infections with CAstV. In addition a flock may be infected with more than one strain of CAstV concurrently.

**Figure 1:**
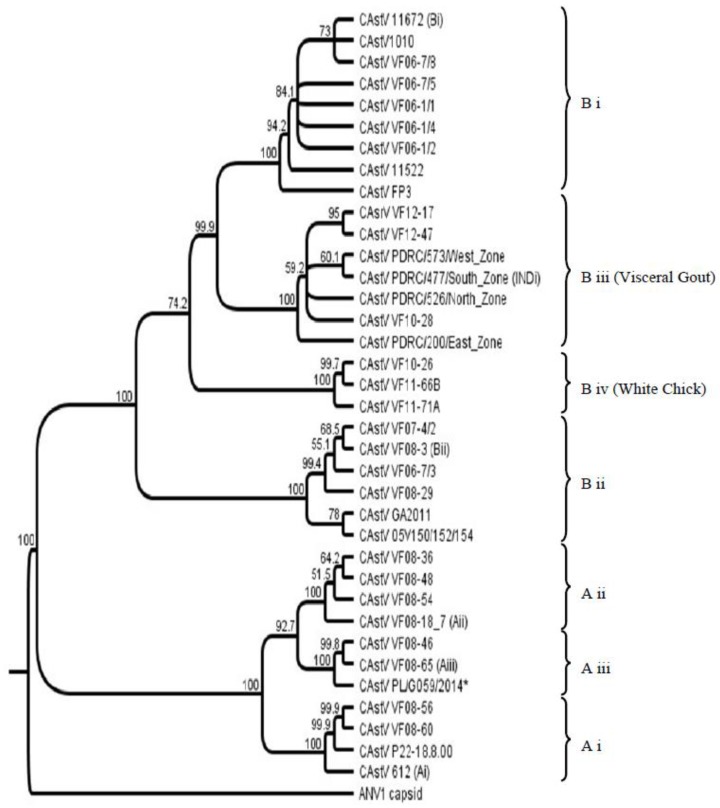
Chicken astrovirus (CAstV) open reading frame (ORF) 2 amino acid sequence. Phylogenetic tree of CAstVs based on complete ORF 2 (capsid) amino acid sequences. The tree was constructed using Geneious v6.1.8 (Biomatters, Auckland, New Zealand) using the neighbor-joining method and 1000 bootstrap replicates (bootstrap values are shown on the tree). Avian nephritis virus serotype 1 (ANV-1) was used to root the tree. * Denotes the Polish CAstV strain associated with White Chicks.

An investigation into CAstV strain diversity from historical and circulating field strains was reported in 2012 comparing sequences of ORF 2 (capsid gene) as this is where the most hypervariable regions associated with antigenicity are located [22]. Prior to this study two distinct serogroups of CAstV had been identified [17,23] which is supported by only a minor degree of cross-reactivity with the heterologous antisera. Antibody against them is reported to be widespread [24] and the existence of these two serogroups was further supported in the genotyping study by the subsequent clustering of strains into CAstV groups A and B according to a lower level of shared amino acid identity across ORF 2 (38%–40%). The group A CAstVs comprised three subgroups, withinter-subgroup homologies from 77% to 82%. The B group CAstVs comprised two subgroups, B i and B ii, which shared inter-subgroup identities of 84%–85% [22]. Subsequent to this study new CAstV ORF 2 amino acid sequences associated with specific broiler chick diseases, namely kidney disease with visceral gout, and White Chicks, have become available and a selection have been incorporated into the CAstV ORF 2 amino acid phylogenetic tree (Figure 1) to help elucidate the association of specific strains with disease.

## 4. Pathogenesis of Chicken Astrovirus

### 4.1. Runting Stunting Syndrome and Uneven Flock Performance

Historically, CAstV has been associated with malabsorption diseases of broiler chickens such as runting stunting syndrome (RSS) and with enteritis and growth problems in flocks [25,26]. However, CAstV is one of a number of endemic, enteric viruses that have been implicated in RSS but as yet a single etiological agent has not been identified. A runted chick hatches small while a stunted bird exhibits a failure to grow, and often appears to have delayed development, where its overall appearance appears to be that of a much younger chick with down and immature feathering, yellow colouration and small comb and beak. RSS is a production disease that was originally characterised by poor weight gain in young broiler flocks, frequently observed between six and twelve days post hatch but can be evident up to three weeks [27]. This coincides with the occurrence of intestinal cysts that reduce nutrient absorption along with reduced villus size or altered villus shape. Other common symptoms include enteritis and diarrhea, leg weakness and irregular feathering [28]. Chicks may huddle for warmth and culling can be extensive due to severe growth check of >50% causing a major economic challenge.

Uneven flock performance occurs when the variance in weights at slaughter is larger than expected, potentially causing carcass processing problems, and is a more common, chronic condition than RSS. Many of the same viruses, including CAstV, are present in underperforming flocks but they are often also present in the good performing flocks so the differences in factors that tip the balance of performance are likely to be subtle yet complex and probably involve co-infection with other pathogens especially other viruses, pathogen strain variation, infection timing, virus load and the presence or absence of maternal antibodies. It is also possible that early CAstV and other enteric viral infections may create an abnormal gut environment that facilitates later dysbacteriosis, an imbalance of naturally colonising bacteria, usually occurring between days 20 and 30 post hatch and which could further impair performance due to diminished nutrient digestibility and weakened intestinal barrier protection [29]. As CAstV and other enteric viruses are so common and widespread in commercial broiler flocks of all classes of performance, they may be considered as part of the normal microflora or gut virome forming a background “noise” that is present from hatch to slaughter in a similar way to the many species of bacteria that colonise poultry intestines. However, it is likely to be a dynamic virome with spatial and temporal changes of rapidly evolving RNA viruses and so infections by new strains or more pathogenic strains of viruses may tip the normal balance of the virome thereby impairing performance or in acute cases causing RSS.

CAstV is one of the earliest viruses to infect chicks often in the embryo, when immunity is least developed, and in recent quantitative molecular surveys broiler chicks that have just hatched were found to be shedding very high levels of CAstV, often substantially higher than CAstV infection peaks from chicks that became infected horizontally soon after hatch [21] suggesting that age confers resistance to infection. There are many strains of CAstV in circulation for which pathogenicity is unknown and it is unknown whether all CAstV strains are able to infect vertically. Currently CAstV pathogenicity is determined empirically through challenge experiments but vertical transmission has not been examined by these means. Challenge experiments of day old commercial broiler or SPF chicks with isolated CAstV strains have resulted in varying degrees of growth suppression [17] that is typical of the wide-ranging pathogenicities of viruses with RNA genomes. Furthermore, inoculations with CAstV isolates have not caused the full growth restriction typically observed in cases of RSS when young broiler birds may be <50% of their expected weight at 2–3 weeks old suggesting that there may also be other agents or other factors involved. Pathogenicity studies in specific pathogen free (SPF) chicks of two of the 25 genotyped strains, CAstV 612, which typifies group A, and CAstV FP3, representing group B, detected both viruses in the duodenum, jejunum and ileum, as well as the colorectum [30]. Both viruses were also detected in the liver, kidney and spleen. While the effects of CAstV 612 were relatively mild, CAstV FP3 resulted in intestinal lesions at day 1 post infection (p.i.) that altered the villus to crypt ratio observed at days 3 and 6 p.i. and a prolonged infection of the kidneys from day 1 to day 8 with lesions reportedly more severe than those seen in birds inoculated with ANV-1 (ANV serogroup 1 strain G4260) in the same study [30].

Co-infections of CAstV with other enteric viruses have been observed, most noticeably rotavirus [25,31] and ANV [32] and a multiplex RT-PCR test was designed to detect and distinguish CAstV, ANV and rotavirus from samples simultaneously [33]. More recently the use of viral metagenomics has demonstrated the presence of a wide range of enteric virus families in normal broilers [34] and in growth problem flocks of which *Astroviridae* was one of the more abundant families [35,36]. In a small set of five 3-week old broilers with RSS and two normal broilers of the same age CAstV was only detected in the RSS-affected birds [36]. This contrasts with findings from the Day study [35] where CAstV was detected in SPF control birds. These types of studies are preliminary and few in number but metagenomics is likely to be a powerful diagnostic tool for investigating RSS in the future although it will be important to sample broilers as soon as the growth problems become apparent in order to fully appreciate the role of very early viruses such as CAstV.

### 4.2. Kidney Disease and Visceral Gout

While CAstV is predominantly an enteric virus contracted through the fecal–oral route it is also known to infect organs outside of the enteric tract including the liver and kidneys. Severe kidney disease of young broiler chicks with outbreaks of visceral gout and up to 40% mortality were reported in India in 2012 with the causative agent being identified as a group B CAstV [37]. This particular strain of CAstV was isolated in embryonated SPF chicken eggs using homogenates from 18 CAstV positive kidney samples resulting in significant embryo stunting, liver necrosis and pale, swollen kidneys. Isolates made from clinical kidney homogenates passed through either SPF chicks or SPF eggs and inoculated into day old SPF chicks and day old broiler chicks resulted in extremely high levels of mortality (67.5%–100%) between days 5 and 10 p.i. for the SPF chicks and days 7 and 10 p.i. for the broilers. Post mortem findings showed that the chicks all had diseased kidneys and visceral gout. Molecular testing found the kidneys positive for CAstV and negative for ANV and infectious bronchitis virus [37].

Phylogenetic analysis of the ORF 2 amino acid sequences from these 18 isolates indicated that there was a high degree of similarity between these strains (92%–99.2%) and that they clustered together and in a separate B subgroup from other CAstV ORF 2 amino acid sequences (Figure 1) [37]. Similarly, AFBI’s Stormont laboratory isolated three highly similar CAstV strains from broiler kidney diagnostic samples from the Middle East as part of a diagnostic investigation into high mortality problems associated with kidney disease and visceral gout in 2010 and 2012, that, when inoculated into day old SPF chicks, also caused mortality due to kidney disease and visceral gout in the first week post infection (diagnostic results). Sequencing and phylogenetic analysis of these strains placed them in the same B subgroup as the Indian strains (B iii, Figure 1). The three Middle East CAstV strains share >99% amino acid homology with each other and 96.5% to 98.8% with regional representative strains from India. Given the wide range of circulating CAstV strains detected previously in Europe and the USA [22] the detection of CAstV strains in India and the Middle East with such a high degree of capsid amino acid conservation in these cases of severe kidney disease supports the hypothesis that this particular strain of CAstV is the etiological agent. Ongoing diagnostic surveillance in 2016 indicates highly similar strains are still circulating in broiler flocks in the Middle East.

### 4.3. White Chicks Hatchery Disease

Recently CAstV has become associated with hatchery diseases, most noticeably “White Chicks”, reports of which have come from various Scandanavian countries, North America, Poland and Brazil [38,39,40], but also with the “clubbed down” problem, although the latter association is less clear and still to be fully determined. White chicks that hatch have pale plumage, are weak and runted and do not tend to survive very long. The symptoms and lesions observed in white chicks share characteristics with those of RSS including lesions in the kidneys and liver, runting/poor development and weakness, and also abnormal feathering. An increase in mid to late embryo deaths was noted and there is a transient but substantial reduction in hatchability, which in Finland averaged 29% in affected flocks but which reached as high as 68% on one farm, with many dead in shell embryos in which CAstV was detected [38]. In Poland, a 4%–5% hatchability decrease was observed for a single breeder flock over a 4-week period when a maximum of 1% of chicks were pale and weak [40]. These observations are indicative of a vertical virus transmission and since it was reported that affected Finnish breeder flocks only experienced the disease once during their lifetime, it seems probable that acquired immunity prevents disease recurrence and further vertical transmission. It was discovered through CAstV quantitative diagnostic testing that many chicks were shedding high loads of CAstV at hatch [38] which was a very different situation to that observed when the same assay was first applied to commercial flocks in 2010 [32]; then all chicks were negative for CAstV at day 0 in a longitudinal survey of commercial broiler flocks.

Three CAstV isolates were purified from samples from Finland, Norway and Canada resulting in embryo death and runting when inoculated into SPF chick embryos [38]. Likewise the Polish PL/G059/2014 isolate caused high mortality, runting and poor hatchability when inoculated into SPF embryonated eggs [40]. When the amino acid sequences of the ORF 2 regions were compared the Scandinavian and Canadian isolates were highly similar, sharing 95%–98% identity, which is a significantly high level of conservation in the most variable CAstV ORF given the wide range of CAstV strains in circulation that can vary by more than 50% in this ORF. These strains clustered together giving rise to a new CAstV B subgroup (B iv, Figure 1) strongly suggestive that strains with highly similar capsid gene sequences could cause hatchery disease. They were also quite highly related to the CAstV strains responsible for severe kidney disease in Asia constituting subgroup B iii with shared amino acid identities ranging from 86.5% to 89.8%. By contrast, the ORF 2 amino acid sequence of the Polish strain, PL/G059/2014, places it very distant in subgroup A iii (Figure 1, marked with an asterisk). Similar symptoms were apparent from the Polish case but the clinical outcomes were less pronounced: there was no perceived egg drop; the hatchability reduction was much less severe and there were fewer white chicks observed than in the Finnish cases. Perhaps the differences in White Chicks disease severity are associated with genomic differences, although a more in-depth analysis of further cases would be necessary to establish the link between specific strain variation and disease severity.

## 5. Immunity, Treatments and Future Developments

Currently there are no medicines to treat RSS, CAstV-associated kidney disease or White Chicks disease nor are there any vaccines to prevent transmission of CAstV to broiler chicks. Hygiene and biosecurity are the only ways in which CAstV infection risk can be minimised. It would be highly advantageous if breeder hens could supply adequate CAstV maternal antibodies to the eggs since this would prevent vertical transmission of CAstV strains and give early protection against horizontal transmission. Although it has yet to be definitively determined, the age of the chick when first infected appears to have a bearing on all of these conditions so early protection is encouraged. Breeder hens that become naturally CAstV seropositive during rear or through the use of a CAstV breeder vaccine is advocated in order to protect embryos and hatched chicks against the range of CAstV strains in circulation and prevent vertically infected hatched chicks from shedding CAstV to infect naive broiler chick housemates. While the involvement of CAstV as a key agent in cases of RSS remains to be fully elucidated, it is clear from recent evidence that certain strains of CAstV are associated with White Chicks and others with severe kidney disease and visceral gout. The development of a commercial vaccine that can protect against the strains causing these two diseases, which are not that far apart genetically and serologically is to be hoped.

The use of wild-type strains of CAstV as breeder vaccine candidates that can be conveniently grown in eggs or cell culture has the advantage of cellular replication to higher titres but may be limited in effectiveness due to serological differences between strains and so a greater understanding of the relationship between circulating strain diversity and disease severity is desirable, particularly in the case of RSS. There is also the concern that an attenuated live vaccine could evolve into a more pathogenic form, although this is unlikely to unduly affect older birds. Alternative vaccine strategies may involve the use of recombinant protein technology to develop non-replicative CAstV capsid precursor proteins. Recombinant CAstV capsids have been produced by two groups using the baculovirus system, to subgroup B ii [41] and subgroup B i [42]. The recombinant CAstV B ii vaccine gave partial protection against experimental RSS challenge whereby weight restriction was significantly less pronounced in vaccinated broiler chicks [41]. Lee *et al.* demonstrated that the B i recombinant CAstV capsid precursor proteins consistently stimulated virus-specific antibodies in SPF chickens at 3 and 4 weeks p.i. after 2 immunizations [42]. Given that wild-type infections of breeder birds with the White Chicks strain of CAstV appear to confer lifetime immunity, it is hoped that an effective CAstV vaccine would have the same duration of effect but this can only be determined empirically.

One of the limitations of working with CAstV has been a lack of convenient diagnostic tools requiring researchers to develop their own in-house tools. The baculovirus expressed recombinant CAstV capsid precursor proteins developed as vaccine candidates have both been used successfully in ELISA (enzyme linked immunosorbent assay) tests to quantify CAstV seroconversion during *in vivo* CAstV experiments [41,42]. The B i recombinant capsid protein has since been used as the basis of a CAstV B group ELISA test [43] that is now commercially available and suitable for screening chicken sera for the presence of CAstV B group antibodies, including those from the other B subgroups. This ELISA is useful for screening breeder flocks for seroconversion against CAstV B group strains prior to, or during lay, and can be used to pinpoint CAstV B group seroconversion by longitudinal serological surveys in cases of possible vertical transmission, e.g., White Chicks. It will not detect antibodies to CAstV A group strains as there is no serological cross reactivity of the A group and B group antibodies in the capsid precursor protein region. If further evidence appears that substantiates the involvement of CAstV A group strains in cases of White Chicks, then a similar CAstV A group ELISA would prove beneficial.

## Figures and Tables

**Table 1:** Avian Astrovirus Species and Associated Diseases.

Avian Species	Virus	Disease/Condition	Major Tissue Distribution
Turkey (ICTV designation: *Avastrovirus 1*)	Turkey astrovirus type 1 (TAstV-1)	Enteritis, growth retardation	Intestine
	Turkey astrovirus type 2 (TAstV-2)	Enteritis, growth retardation, (PEC: poult enteritis complex)	Intestine, bursa of Fabricius, thymus
Chicken (ICTV designation: *Avastrovirus 2*)	Avian nephritis virus (ANV)	Nephritis, baby chick nephropathy, growth retardation	Intestine, kidney
	Chicken astrovirus (CAstV)	Growth retardation, kidney disease, White Chicks hatchery disease	Intestine, kidney, liver pancreas, spleen
Duck (ICTV designation: *Avastrovirus 3*)	Duck astrovirus type 1 (DAstV-1)	Hepatitis and variable mortality in young ducks	Liver, kidney, spleen
	Duck astrovirus type 2 (DAstV-2)	Hepatitis and variable mortality in ducklings	Liver, kidney, spleen
